# A Pathophysiologically Hypertrophic 3T3-L1 Cell Model—An Alternative to Primary Cells Isolated from DIO Mice

**DOI:** 10.3390/cells14110837

**Published:** 2025-06-03

**Authors:** Isabell Kaczmarek, Kristiana Schüßler, Andreas Lindhorst, Martin Gericke, Doreen Thor

**Affiliations:** 1 Rudolf Schönheimer Institute of Biochemistry, Medical Faculty, Leipzig University, 04103 Leipzig, Germany; isabell.kaczmarek@medizin.uni-leipzig.de (I.K.);; 2 Institute of Anatomy, Medical Faculty, Leipzig University, 04103 Leipzig, Germany; andreas.lindhorst@medizin.uni-leipzig.de (A.L.); martin.gericke@medizin.uni-leipzig.de (M.G.)

**Keywords:** obesity, adipose tissue, adipocyte hypertrophy, cell model

## Abstract

Adipocyte hypertrophy in individuals with obesity is connected to alterations in adipocyte function. These pathophysiological changes are studied using animal models and adipose tissue engineering. However, knockdown, overexpression, and stimulation studies would benefit from an easily applicable cell model. Although several models (free fatty acids, glucose restriction, and long-term incubation) have previously been described, our evaluation demonstrated that they lack important features described for hypertrophic adipocytes found in obesity. Therefore, we aimed to develop a cell model depicting the pathophysiological state of adipocytes in obesity by applying novel approaches (insulin, macrophage supernatant, and Tnfα) using 3T3-L1 cells. To analyze changes in adipocyte phenotype and function, we detected the cell size, lipid accumulation, insulin sensitivity, cytokine/adipokine secretion, and expression of lipolytic enzymes. Combining long-term incubation with insulin and Tnfα co-stimulation, we found significantly increased cell size and lipid accumulation compared to 3T3-L1 adipocytes differentiated with standard protocols. Furthermore, these adipocytes showed significantly reduced insulin sensitivity, adiponectin secretion, and lipolytic enzyme expression, accompanied by increased IL6 and leptin secretion. In summary, the described cell model depicts pathophysiologically hypertrophic 3T3-L1 adipocytes. This model can be used for knockdown, overexpression, and stimulation studies, thereby serving as an alternative to primary cells isolated from DIO mice.

## 1. Introduction

The prevalence of obesity is increasing worldwide, affecting more than one billion people [1,2]. The associated negative systemic health effects lead to comorbidities, like type 2 diabetes [2,3,4,5,6], resulting in a high demand for obesity prevention and treatment.

In obesity, an imbalance between energy intake and energy expenditure leads to excessive lipid accumulation in adipose tissue (AT). In particular, excessive expansion of visceral AT is a predictor for metabolically unhealthy obesity [7,8,9]. The expansion of AT in obesity shifts from a balance between hypertrophy and hyperplasia of (pre)adipocytes towards hypertrophy [10,11]. This shift is connected to altered hormone secretion and reduced insulin sensitivity of adipocytes [11,12,13,14]. Changes in adipocyte function that occur in a pathologically hypertrophic state, like obesity, are currently investigated using animal models, organ chips, or hypertrophic adipocyte cell lines.

Animal models, mostly mice and rats, are widely used in obesity research. Obesity can be induced in animal models by various factors [15]; the use of a high-calorie diet (e.g., a high-fat diet) is a common method [16,17]. From these models, primary adipocytes can be isolated to analyze their hypertrophic state ex vivo. Compared to the use of human subjects, animal models benefit from genetic identity, standardized experimental conditions, and genetic manipulability. However, feeding an unphysiological diet and the associated excessive lipid accumulation place a high burden on these animals.

AT engineering is of increasing importance in basic and translational research. In this approach, isolated precursor cells are differentiated into mature adipocytes in the presence of matrix proteins [18]. Improvements in these initial models have led to the development of AT organ chips, which mimic the conditions found in human tissue three-dimensionally [19] and can be used for functional investigations [20,21]. To analyze hypertrophic adipocytes from individuals with obesity directly and to circumvent the differentiation of precursor cells ex vivo, a chip system using primary human subcutaneous adipocytes was invented [22]. Nevertheless, its poor accessibility and low transfectability render this approach unsuitable for basic research. Therefore, cell culture models with adipogenic potential remain important for obesity research.

Several cell lines with adipogenic potential are available for obesity research [23]. For some of these cell lines, hypertrophic models have been established to map the characteristics of adipocytes from individuals with obesity. Since excessive lipid accumulation in the AT of individuals with obesity increases free fatty acids (FFAs), which are responsible for hypertrophy and insulin resistance in adipocytes [24], mature adipocytes have been stimulated with palmitate and oleate to induce hypertrophy [25,26,27]. Another model induced hypertrophy in adipocytes by permanent cultivation under high glucose (25 mM) in comparison to reduced glucose (5.55 mM) concentrations [28]. Furthermore, the long-term cultivation of mature adipocytes was described to lead to increased lipid accumulation and altered gene expression, similarly to what is observed in individuals with obesity [29].

Interestingly, the hypertrophic phenotype of these models has only been partially analyzed and comparative and comprehensive studies are lacking. Therefore, we evaluated published and new approaches and optimized the most promising method to generate a suitable hypertrophic adipocyte cell model using 3T3-L1, a mouse cell line with adipogenic potential.

## 2. Materials and Methods

### 2.1. Cell Culture

3T3-L1 CL-173™ cells, purchased from ATCC (Manassas, VA, USA), were cultured and differentiated according to established protocols [30]. In brief, culture medium (CM, DMEM (Thermo Fisher Scientific, Waltham, MA, USA) supplemented with 10% FBS (Thermo Fisher Scientific, Waltham, MA, USA), 100 U/mL penicillin (Thermo Fisher Scientific, Waltham, MA, USA), and 100 µg/mL streptomycin (Thermo Fisher Scientific, Waltham, MA, USA)) was used for standard 3T3-L1 cultivation at 37 °C in a humidified atmosphere (5% CO_2_). Before the cells reached confluence, splitting was performed, and the cells were seeded at a density of 100,000 to 200,000 cells per T175 flask (Greiner Bio-One, Kremsmünster, Austria). To obtain mature adipocytes, the 3T3-L1 cells were differentiated. Thereto, the cells were grown until confluence (D-2) in CM. After another two days, the CM was exchanged with differentiation medium 1 (DM1, CM containing 0.172 mM insulin (Sigma-Aldrich, St. Louis, MO, USA), 0.25 µM dexamethasone (Sigma-Aldrich, St. Louis, MO, USA), 0.5 mM IBMX (Sigma-Aldrich, St. Loius, MO, USA), 2 µM rosiglitazone (Sigma-Aldrich, St. Louis, MO, USA), D0). After a three-day incubation (D3), DM1 was replaced by differentiation media 2 (DM2, CM containing 0.172 mM insulin), and the cells were incubated for another three days. Subsequently, the medium was changed to CM every other day (D6, D8). On day 10 (D10), standard differentiation into mature adipocytes was complete.

THP1 cells were cultivated in macrophage culture medium (RPMI-1640 (Gibco, Waltham, MA, USA)) supplemented with 10% heat-inactivated (HI, 30 min at 56 °C) FBS, 100 U/mL penicillin, and 100 µg/mL streptomycin) and split every 3–4 days (4,000,000 cells/T75-flask (Greiner Bio-One, Kremsmünster, Austria) in 20 mL). The THP-1 cells were split into 12-well plates at a density of 500,000 cells/well. To obtain M0 macrophages, macrophage culture medium containing 50 ng/mL PMA (Sigma-Aldrich, St. Louis, MO, USA) was added for 48 h. Polarization into M1- or M2-like macrophages was achieved by subsequent incubation for another 48 h in macrophage culture medium mixed with either 20 ng/mL LPS (Sigma-Aldrich, St. Louis, MO, USA) and 20 ng/mL IFNγ (Sigma-Aldrich, St. Louis, MO, USA) (M1) or 25 ng/mL IL-4 (Sigma-Aldrich, St. Louis, MO, USA) and 25 ng/mL IL-13 (Sigma-Aldrich, St. Louis, MO, USA) (M2), as previously described [31]. After polarization of the THP-1 cells into M0, M1, and M2 macrophages, the cells were equilibrated to adipocyte culture medium for 24 h. Subsequently, the THP-1 macrophages were incubated with 500 µL/well adipocyte culture medium for 24 h. The supernatant was pooled and centrifuged (5 min, 200× *g*) to remove the cells before snap freezing.

### 2.2. Generation of Hypertrophic 3T3-L1 Adipocyte Models

**Figure 1:**
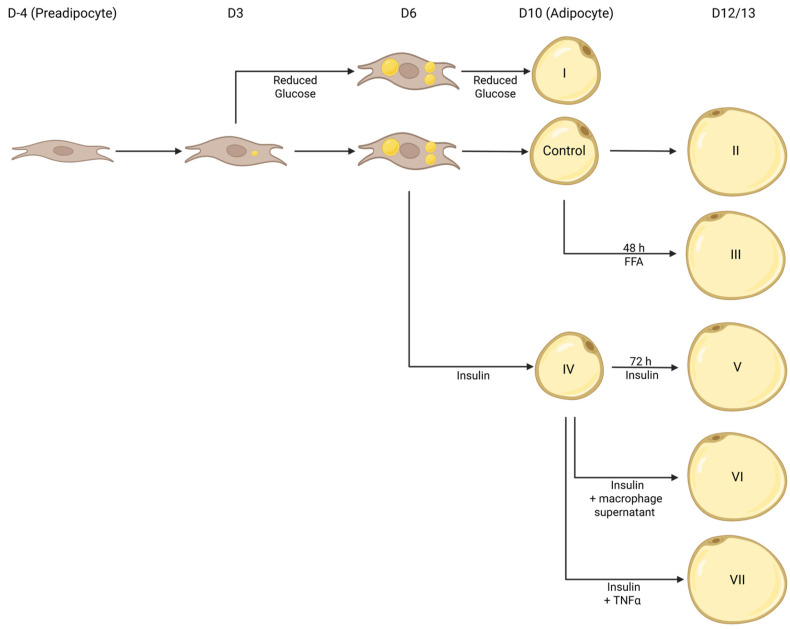
Depiction of (patho)physiologically hypertrophic 3T3-L1 cell models. Using various approaches (I: Reduced Glucose; II: long-term incubation; III: FFAs; IV: Insulin; V: Long-term Incubation + Insulin; VI: long-term incubation + Insulin + M0, M1, or M2 THP1 macrophage supernatant; VII: long-term incubation + Insulin + Tnfα), 3T3-L1 cells were induced to become (patho)physiologically hypertrophic. Created in Biorender. Thor, D. (2025) https://BioRender.com/l205457 (accessed on 1 May 2025).

The (patho)physiologically hypertrophic models were generated according to the differentiation protocol shown in Figure 1 and Table 1 and compared to standard differentiated 3T3-L1 (D10 *w/o*) described in Section 2.1.

### 2.3. Differential Gene Expression of Marker Genes

Gene expression analysis of RNAseq data was conducted using FATTLAS [32]. In brief, expression data of mouse visceral AT (dataset #1: GSE91067; dataset #2: GSE132706), visceral adipocytes (vAdi, GSE129665, GSE168906), and associated annotations were downloaded before plotting and significance testing between lean individuals and individuals with obesity.

### 2.4. Expression Analysis Using Quantitative PCR

3T3-L1 cells were washed using PBS (Thermo Fisher Scientific, Waltham, MA, USA), harvested, and stored at −80 °C until RNA was isolated following the manufacturer’s protocol (ReliaPrep™ RNA Miniprep Systems, Promega, Madison, WI, USA). Reverse transcription was performed using SuperScript II™ (Thermo Fisher Scientific, Waltham, MA, USA). Quantitative PCR was performed by combining 10 ng of cDNA and 1.2 µM of primer mix with either Platinum^®^ SYBR-Green qPCR SuperMix-UDG (Thermo Fisher Scientific, Waltham, MA, USA) or Luna Universal qPCR Master Mix (BioRad, Hercules, CA, USA). Real-time PCR measurements were acquired with the CFX Connect™ Real-Time PCR Detection System (Bio-Rad, Hercules, CA, USA). Data were normalized to β-actin (*Actb*), which served as recommended housekeeping gene [33] (ΔCt), and to standard differentiated adipocytes (D10 *w/o*, ΔΔCt). Primers were designed using NCBI primer blast [34] and synthesized by Microsynth (Balgach, Switzerland). Only primers with a duplication efficiency within the range of E = 1.9–2.1 were selected for analysis. Sequences of validated primers are given in Supplementary Table S1.

### 2.5. Protein Isolation, SDS-PAGE, and Western Blot

Cells were washed with PBS before lysis in RIPA buffer (150 mM NaCl (Carl Roth, Karlsruhe, Germany), 10 mM Tris (pH 7.2, Carl Roth, Karlsruhe, Germany), 0.1% SDS (BioRad, Hercules, CA, USA), 1% Triton X-100 (Sigma-Aldrich, St. Louis, MO, USA), 1% Deoxycholate (Sigma-Aldrich, St. Louis, MO, USA), 5 mM EDTA (Carl Roth, Karlsruhe, Germany), 200 µM PMSF (Sigma-Aldrich, St. Louis, MO, USA), 1 mM Na-Orthovanadate (Sigma-Aldrich, St. Louis, MO, USA), Halt™ Protease and Phosphatase Inhibitor Cocktail (Thermo Fisher Scientific, Waltham, MA, USA)). After vortexing, ultrasonic treatment (3 × 15 s), and a 60 min incubation at 4 °C while gently shaking, the lysate was centrifuged (14,000× *g*, 4 °C, and 15 min). Protein concentration in the aqueous phase was determined with the Pierce™ BCA Protein Assay Kit (Thermo Fisher Scientific, Waltham, MA, USA). Then, 50 µg of protein was separated by SDS-PAGE before Western blotting using the semidry blot method. The following antibodies and dilutions were used for immunodetection: anti-β-Actin (1:1000, #A1978, Sigma-Aldrich, St. Louis, MO, USA), anti-HSL (1:1000, #4107, CST, Danvers, MA, USA), anti-PLIN1 (1:1000, #ab3526, Abcam, Cambridge, UK), anti-ATGL (1:1000, #2138, CST, Danvers, MA, USA), anti-rabbit-IgG-HRP (1:5000, #7074, CST, Danvers, MA, USA), and anti-mouse-IgG-HRP (1:5000, #7076, CST, Danvers, MA, USA).

### 2.6. Lipid Droplet Staining and Droplet Analysis

Following the differentiation of 3T3-L1 fibroblasts into mature adipocytes and the treatments (described in Section 2.1 and Section 2.2), cells were fixed in a two-step protocol using 10% formaldehyde/PBS (5 min and 1 h, Carl Roth, Karlsruhe, Germany) before washing with 60% isopropanol (Carl Roth, Karlsruhe, Germany). To prepare the Oil Red O (ORO, Sigma-Aldrich, St. Louis, MO, USA) stock solution, 3.5 g/L ORO was dissolved in isopropanol and stored at 4 °C. Prior to use, a fresh working solution was obtained by diluting the stock solution with deionized water in a 60:40 ratio, incubating at room temperature for 20 min, and filtering (0.2 µm mesh, Sarstedt, Nümbrecht, Germany), which resulted in a final concentration of 2.1 g/L ORO. Fixed cells were stained with the freshly prepared ORO working solution for 10 min. After incubation, cells were promptly washed four times with tap water. Images were captured using Keyence bz-x800_long (Keyence, Osaka, Japan) and subsequently analyzed with ImageJ (ImageJ 1.53q, Fiji, NIH, Bethesda, MD, USA) [30,35]. ORO was eluted by adding isopropanol and gentle pipetting. OD values were determined at 500 nm and 620 nm using the Sunrise™ photometer (Tecan, Männedorf, Switzerland).

### 2.7. Cell Size Determination

Cell size was analyzed in 3T3-L1 adipocytes after staining with 1:1000 cell mask deep red (Thermo Fisher Scientific, Waltham, MA, USA). Fluorescence images were taken with the Olympus fluoview 1000 (Shinjuku, Japan). The adipocyte cell membranes were manually drawn onto the exported images, and the resulting perimeters were filled out (Sketchbook, San Rafael, CA, USA). After exporting a separate image containing all adipocyte masks of the respective fluorescence image, cell size was determined using ImageJ.

### 2.8. Analysis of Cell Number and Viability

To stain 3T3-L1 adipocytes for necrosis and analyze cell count, cells were stained with Propidium-iodide (1 μg/mL, Sigma-Aldrich, St. Louis, MO, USA) and Hoechst33342 (1 μg/mL, Sigma-Aldrich, St. Louis, MO, USA) for 20 min prior to image acquisition and analysis with the Celigo Image Cytometer (Nexcelom Bioscience, Lawrance, MA, USA). Apoptosis was assessed using the Apo-ONE^®^ Homogeneous Caspase-3/7 Assay (Promega, Madison, WI, USA) following the manufacturer’s instructions.

### 2.9. Analysis of Adipocyte Function

The secretion of leptin, adiponectin, and IL6 was analyzed in the supernatant from 3T3-L1 adipocytes, which were kept serum-free overnight before 24 h stimulation in serum-free media. Additional supplements and acute stimulations depending on the model system are described in the corresponding figure legends. To determine the total amount of secreted protein in the supernatant and secretion rate, a mouse leptin ELISA (CrystalChem, Elk Grove Village, IL, USA), a mouse adiponectin ELISA (Thermo Fisher Scientific, Waltham, MA, USA), and a mouse IL6 AlphaLISA (PerkinElmer, Waltham, MA, USA) were used, and the results were normalized to the cell number.

AKT phosphorylation was detected in mature 3T3-L1 cells. Therefore, cells were serum-, insulin-, and Tnfα-starved for 2 h before acute insulin signaling was induced by adding 100 nM of insulin for 15 min. Following this, cells were washed with PBS before measuring AKT phosphorylation and total AKT using Alpha SureFire Ultra Multiplex p-AKT1/2/3(Ser473)/Total AKT1 (PerkinElmer, Waltham, MA, USA).

Glucose uptake was measured in fully differentiated 3T3-L1 cells using [^3^H]-labeled deoxy-D-glucose (Perkin Elmer, Waltham, MA, USA). After 2 h of serum, insulin, and Tnfα starvation, glucose uptake was induced by adding 100 nM of insulin for 15 min prior to adding 0.5 µCi/mL of [^3^H]-2-deoxy-D-glucose (PerkinElmer, Waltham, MA, USA) and 100 µM of 2-deoxy-D-glucose (Sigma-Aldrich, St. Louis, MO, USA) for 30 min. Medium was removed and the cells were washed twice with ice-cold PBS and lysed by RIPA buffer. Glucose uptake was determined from the cell lysate using a scintillation counter (PerkinElmer, Waltham, MA, USA) and normalized to protein content analyzed using the BCA assay.

### 2.10. Quantification and Statistical Analysis

Statistical details of experiments are described in the figure legends. GraphPad Prism software version 10.4.0 (San Diega, CA, USA) was used to complete statistical analysis (Student’s *t*-test and two-way ANOVA).

## 3. Results

### 3.1. Incubation with Insulin Induces Prominently Increased Lipid Accumulation

**Figure 2:**
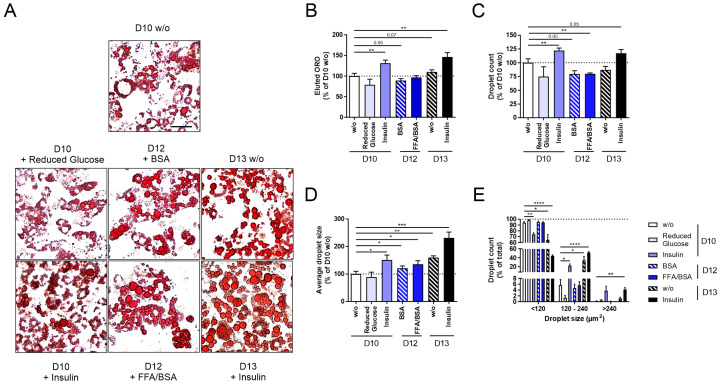
Changes in lipid accumulation of 3T3-L1 adipocytes after glucose restriction, stimulation with free fatty acids (FFAs, only 750 µM palmitate shown) or insulin, or after long-term incubation. 3T3-L1 cells were differentiated under various conditions (glucose restriction, FFA or insulin stimulation, long-term incubation; Figure 1) to induce hypertrophic 3T3-L1 adipocytes. Following this, 3T3-L1 adipocytes were stained with ORO for quantitative and qualitative lipid droplet analysis. As a control, standard differentiated 3T3-L1 cells (D10 *w/o*) were used. (**A**) Microscopic pictures of the applied conditions show differences in lipid accumulation (scale bar: 50 µm). (**B**) For quantification, ORO was eluted from adipocytes and normalized to D10 *w/o* (OD_500 nm–620 nm_ = 0.51 ± 0.03, *n* ≥ 3). For qualitative lipid droplet analysis, (**C**) droplet count (*n* ≥ 3), (**D**) average droplet size (n ≥ 3), and (**E**) lipid droplet size distribution were measured (*n* ≥ 3). Droplet count and average droplet size were normalized to D10 *w/o* (n_droplet_ = 4879 ± 348, A_droplet_ = 28.15 ± 2.81 µm^2^). The mean ± SEM of biological replicates is depicted. Significance was tested using a paired Student’s *t* test (**B**–**D**) or two-way ANOVA (**E**). * *p* < 0.05, ** *p* < 0.01, *** *p* < 0.001, **** *p* < 0.0001.

Adipocytes are well known for their lipid/energy storing and endocrine functions. In individuals with obesity, the expansion of AT is characterized by increased lipid accumulation, mostly driven by adipocyte hypertrophy rather than hyperplasia and adipogenesis, leading to the formation of larger lipid droplets and adipocytes [10,11]. To establish a cell model mimicking this phenotype, we carried out a qualitative and quantitative evaluation of the influence of various treatments (Reduced Glucose, FFAs, Insulin, long-term incubation; Figure 1) [26,27,28,29] on lipid accumulation in comparison to standard differentiated 3T3-L1 adipocytes (D10 *w/o*) using Oil Red O (ORO) (Figure 2).

When investigating adipogenesis under glucose restriction (D10 + Reduced Glucose), we observed slightly reduced lipid accumulation (Figure 2A–E). After differentiating 3T3-L1 cells under constant insulin stimulation (D10 + Insulin), we detected significantly increased lipid accumulation (Figure 2B), droplet number (Figure 2C), and average droplet size (Figure 2D). In particular, the number of small lipid droplets was reduced, while that of middle-sized droplets increased (Figure 2E). After stimulating mature 3T3-L1 adipocytes with FFAs/BSA (D12 + FFAs/BSA) or BSA (D12 + BSA) for two days, we observed a significantly reduced droplet number; however, droplet size increased already for the control-stimulated D12 + BSA cells (Figure 2A–E). No difference was detected between D12 + BSA and D12 + FFAs/BSA (Figure 2A–E), indicating that longer incubation rather than the stimulation with FFAs/BSA lead to a more hypertrophic phenotype.

To maximize the hypertrophic effects, we combined the most promising approaches, insulin and long-term incubation, and investigated lipid accumulation after long-term stimulation with insulin on D13 of differentiation (D13 + Insulin). Here, we found an even more pronounced hypertrophic phenotype compared to insulin stimulation (D10 + Insulin) and long-term incubation (D12 + BSA) alone (Figure 2A–E).

Therefore, we chose the constant insulin stimulation and long-term incubation of 3T3-L1 cells (D10 + Insulin, D13 *w/o*, D13 + Insulin) to proceed with the validation of other characteristics of hypertrophic adipocytes.

### 3.2. Insulin and Long-Term Insulin Stimulation Increase Cell Size and Leptin Secretion

**Figure 3:**
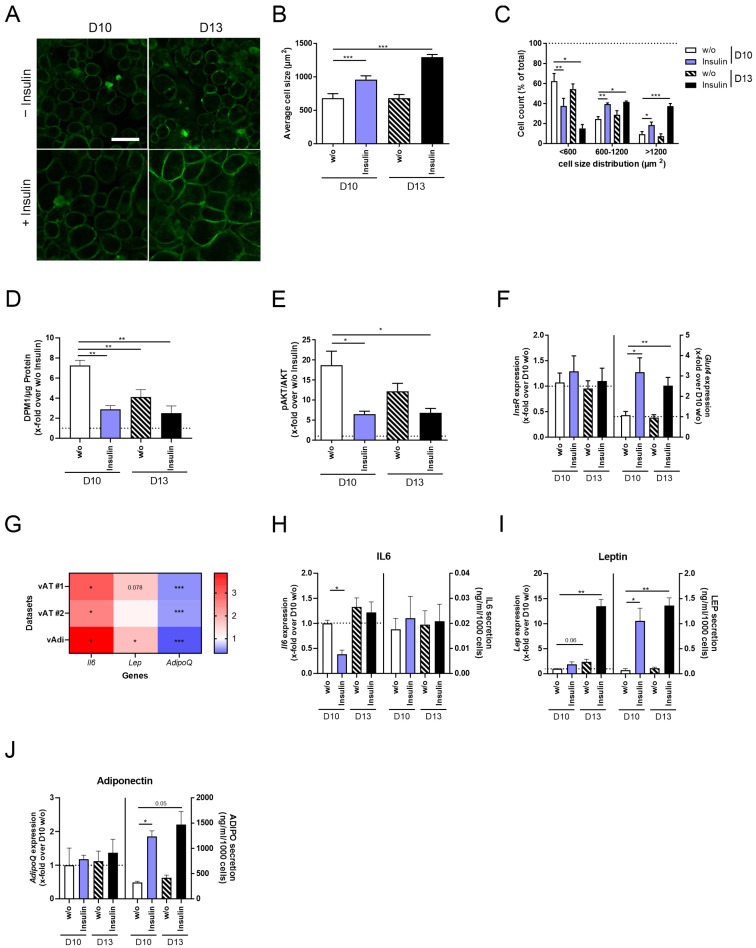
Phenotypical and functional alterations of 3T3-L1 adipocytes after insulin stimulation and/or long-term incubation. 3T3-L1 cells were differentiated into mature adipocytes until D10 or D13 of differentiation, either with the supplementation of insulin from D0 to D6 of differentiation or in the continuous presence of insulin (Figure 1). As a control, standard differentiated 3T3-L1 cells (D10 *w/o*) were used. The plasma membranes of 3T3-L1 cells were stained to (**A**) microscopically visualize variations in cell size (scale bar: 50 µm) and to analyze (**B**) average cell size (*n* = 5) and (**C**) cell size distribution (*n* = 5). When investigating insulin sensitivity, (**D**) acute insulin-stimulated glucose uptake (*n* = 5) and (**E**) p-AKT/AKT (*n* = 6) were detected and normalized to non-stimulated cells. Further, the expression of (**F**) *InsR* and *Glut4* was determined and normalized to D10 *w/o* (*n* = 5). (**G**) To verify AT marker genes for obesity and, therefore, for hypertrophy, the gene expression of the adipokines *Lep* and *AdipoQ* and of the pro-inflammatory cytokine *Il6* was analyzed using FATTLAS [32]. The expected changes in the expression of all three genes were observed in two independent datasets of visceral AT and one dataset of visceral adipocytes. The expression is shown as the ratio of obese/lean. Following this, the gene expression and secretion of (**H**) *Il6*/IL6 (*n* ≥ 3), (**I**) *Lep*/LEP (*n* ≥ 3), and (**J**) *AdipoQ*/ADIPO (*n* ≥ 3) were measured in all conditions. To analyze adipokine secretion, cells were serum-starved overnight followed by stimulation in serum-free media supplemented with insulin. Expression data were normalized to *Actb* and D10 *w/o*, and secretion data were normalized to cell count. The ΔCt and Ct*_Actb_* of (**F**,**H**–**J**) are summarized in Supplementary Table S2. The mean ± SEM of biological replicates is given. Significant changes were tested using a paired Student’s *t* test (**B**,**D**–**J**) and two-way ANOVA (**C**). * *p* < 0.05, ** *p* < 0.01, *** *p* < 0.001.

Besides increased lipid accumulation in adipocytes, hypertrophy, in general, is characterized by an enlarged cell size. Furthermore, obesity-caused adipocyte hypertrophy is additionally associated with reduced insulin sensitivity [11,12,13] and a variation in the expression and secretion of adipokines and cytokines [11,14]. Therefore, we characterized changes in cell size and adipocyte function under constant insulin stimulation and/or long-term incubation in comparison to D10 *w/o* adipocytes (Figure 3).

When investigating phenotypical changes in 3T3-L1 adipocytes, we detected increased cell size induced by insulin stimulation (D10 + Insulin) compared to D10 *w/o* adipocytes, which was even more pronounced in D13 + Insulin cells (Figure 3A,B). Simultaneously, cell size distribution was shifted to a significantly higher number of middle-sized and large adipocytes in D10 + Insulin compared to D10 *w/o* adipocytes, which again was more evident in D13 + Insulin adipocytes (Figure 3C).

The functional analysis of 3T3-L1 adipocytes revealed impaired insulin sensitivity in D10 + Insulin and D13 + Insulin adipocytes, characterized by reduced acute insulin-stimulated glucose uptake (Figure 3D) and AKT phosphorylation (Figure 3E), which was not caused by the dysregulation of *InsR* mRNA levels (Figure 3F, Supplementary Table S2). As a compensatory mechanism, however insufficient, *Glut4* expression was increased (Figure 3F, Supplementary Table S2). For further characterization, we analyzed the expression of three marker genes for obesity, the pro-inflammatory cytokine *Il6* [36,37,38] and the adipokines *Lep* [39,40,41] and *AdipoQ* [42,43], using RNAseq datasets of visceral AT and visceral adipocytes from lean and obese mice [32]. We were able to reproduce the finding that *Il6* and *Lep* are significantly upregulated in the visceral AT and adipocytes of obese mice, while *AdipoQ* was significantly downregulated (Figure 3G, Supplementary Table S2). In D10 + Insulin adipocytes, we detected significantly lower *Il6* expression but no changes in IL6 secretion (Figure 3H, Supplementary Table S2). *Lep* expression was significantly increased in D13 + Insulin adipocytes, accompanied by higher leptin secretion in D10 + Insulin and D13 + Insulin adipocytes (Figure 3I, Supplementary Table S2). The gene expression of *AdipoQ* was constant, whereas adiponectin secretion was significantly induced in D10 + Insulin and D13 + Insulin adipocytes (Figure 3J, Supplementary Table S2).

Evaluating the effect of constant insulin stimulation and long-term incubation on 3T3-L1 adipocytes verified a hypertrophic phenotype for D10 + Insulin adipocytes, which was more evident in D13 + Insulin adipocytes. However, pathophysiologic characteristics like higher IL6 expression and reduced adiponectin secretion were not observed, indicating that our cell model requires further optimization.

### 3.3. Co-Stimulation of Insulin with THP1 Macrophage Supernatant Is Not Sufficient to Induce Pathophysiologic Hypertrophy in 3T3-L1 Adipocytes

**Figure 4:**
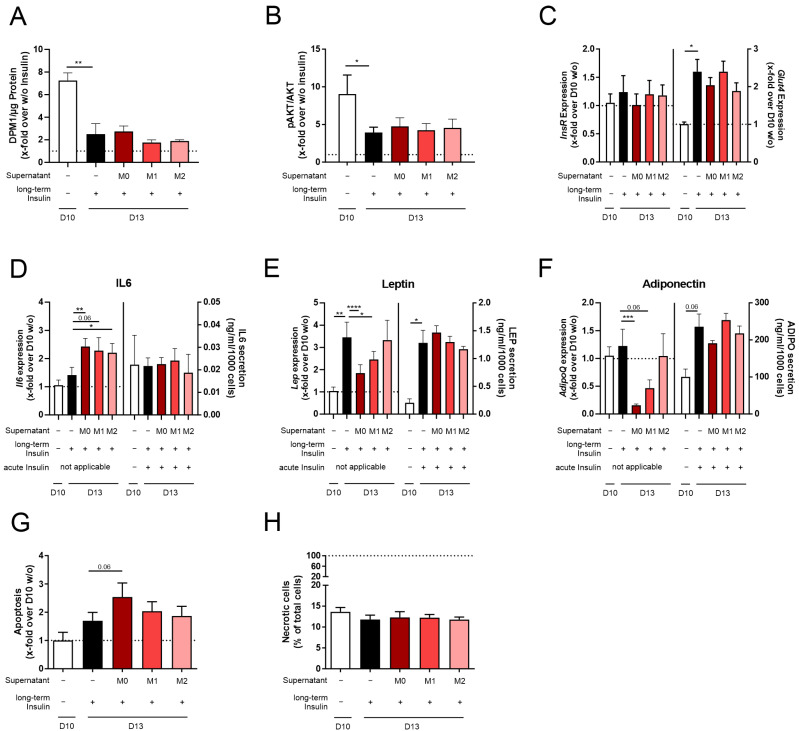
Changes in the adipocyte function and viability of 3T3-L1 adipocytes differentiated with insulin and co-stimulated with insulin and M0, M1, or M2 THP-1 macrophage supernatant. 3T3-L1 adipocytes were differentiated under long-term insulin stimulation until D13, with co-stimulation of M0, M1, or M2 THP-1 macrophage supernatant from D10 to D13 (Figure 1). As a control, standard differentiated 3T3-L1 cells (D10 *w/o*) were used. To investigate acute insulin-stimulated signaling, (**A**) glucose uptake (*n* = 4) and (**B**) p-AKT/AKT (*n* = 7) were measured. Acutely insulin-stimulated cells were normalized against non-stimulated cells. Additionally, the gene expression of (**C**) *InsR* and *Glut4* was detected and normalized to *Actb* and D10 *w/o* (*n* ≥ 3). To investigate adipokine expression and secretion (**D**), *Il6*/IL6 (*n* ≥ 4), (**E**) *Lep*/LEP (*n* ≥ 4), and (**F**) *AdipoQ*/ADIPO (*n* ≥ 4) were analyzed. In the secretion assays, adipocytes were starved and stimulated in serum-free media containing insulin (acute insulin stimulation) as indicated. Expression data were normalized to *Actb* and D10 *w/o*, and secretion data were normalized to cell count. Further, the effect on (**G**) apoptosis and (**H**) necrosis (*n* = 3, compared to Supplementary Figure S1L) was investigated. Apoptosis was normalized to D10 *w/o* (RLU = 1857 ± 533). Necrotic cells were normalized to total cell count. The ΔCt and CT*_Actb_* of (**C**–**F**) are summarized in Supplementary Table S2. The mean ± SEM of biological replicates is depicted, and significance was determined using a paired Student’s *t* test. * *p* < 0.05, ** *p* < 0.01, *** *p* < 0.001, **** *p* < 0.0001.

In individuals with obesity, the number of AT macrophages (ATMs) is increased in comparison to lean individuals [44,45]. Here, the ratio of pro-inflammatory M1 and anti-inflammatory M2 macrophages shifted to M1, leading to a higher inflammatory state of AT [44,45]. Therefore, we decided to co-stimulate insulin-treated 3T3-L1 cells with M0, M1, or M2 macrophage supernatant to investigate the impact of different macrophage subpopulations on the hypertrophic characteristics of D13 + Insulin cells (Figure 1 and Figure 4).

Testing the effect of the macrophage supernatant, we found no additional effect on lipid accumulation (Supplementary Figure S1A–E) and cell size (Supplementary Figure S1F–H) compared to D13 + Insulin cells. Furthermore, acute insulin-stimulated signaling was not influenced compared to D13 + Insulin cells (Figure 4A–C, Supplementary Table S2). *Il6*, *Lep*, and *AdipoQ* expression was significantly altered by the M0, M1, and M2 macrophage supernatants compared to D13 + Insulin cells (Figure 4D–F, Supplementary Table S2). When detecting the effect of acute insulin stimulation on secretion levels, we could not find any difference in IL6, LEP, and ADIPO secretion (Figure 4D–F, Supplementary Figure S1I–K). Here, it needs to be noted that acute stimulation had to be performed in FBS-free media and, therefore, without macrophage supernatant.

When analyzing apoptosis (Figure 4G) and necrosis (Figure 4H, Supplementary Figure S1L) in 3T3-L1 cells co-stimulated with insulin and macrophage supernatant, we did not observe any changes.

Since we could not analyze the acute co-stimulation of 3T3-L1 adipocytes with insulin and M0, M1, or M2 macrophage supernatant in the secretion studies, the effect of macrophages on physiological hypertrophy in adipocytes (D13 + Insulin) could not be detected.

### 3.4. Tnfα Stimulation of Long-Term Insulin-Stimulated 3T3-L1 Adipocytes Further Reduces Insulin Sensitivity and Strengthens Inflammatory State

**Figure 5:**
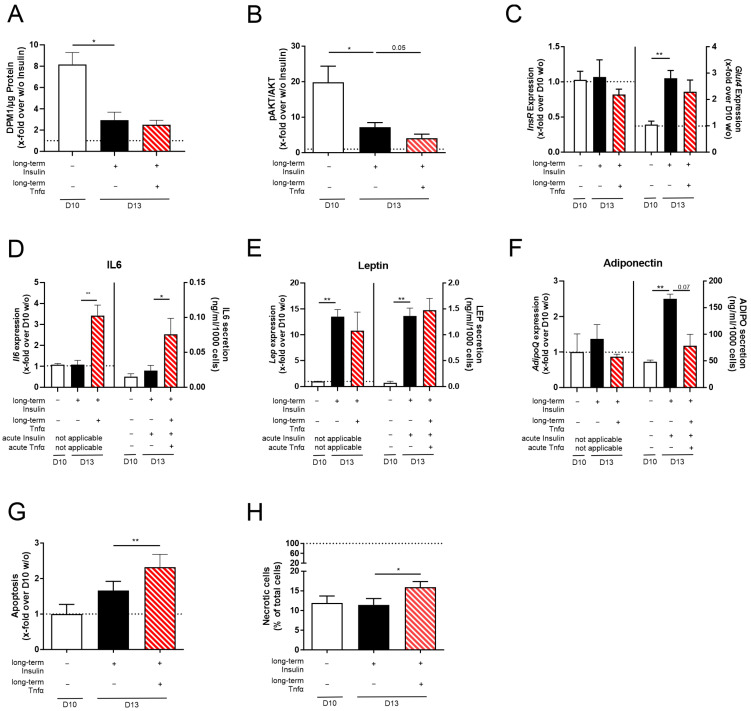
The effect of Tnfα stimulation on the function and viability of long-term insulin-stimulated 3T3-L1 adipocytes. 3T3-L1 adipocytes were differentiated under long-term insulin stimulation until D13 of differentiation, with optional Tnfα stimulation from D10 to D13 (Figure 1). As a control, standard differentiated 3T3-L1 cells (D10 *w/o*) were used. When analyzing acute insulin-stimulated signaling, (**A**) glucose uptake (*n* ≥ 3) and (**B**) pAKT/AKT were determined (*n* = 4). Acute insulin-stimulated cells were normalized to non-stimulated cells. Additionally, (**C**) the expression of *InsR* and *Glut4* was measured (*n* = 5) and normalized to *Actb* and D10 *w/o*. Adipokine gene expression and secretion were detected for (**D**) *Il6*/IL6 (*n* ≥ 3), (**E**) *Lep*/LEP (*n* ≥ 3), and (**F**) *AdipoQ*/ADIPO (*n* ≥ 3). In the secretion assays, adipocytes were starved and stimulated in serum-free media containing insulin (acute insulin) and/or Tnfα (acute Tnfα) as indicated. Corresponding expression values are shown in Supplementary Table S2. Expression was normalized to *Actb* and D10 *w/o*, and secretion data were normalized to cell count. Further, (**G**) apoptosis (*n* = 4) and (**H**) necrosis (*n* = 6, compared to Figure S2L) were measured. Apoptosis was normalized to D10 *w/o* (RLU = 1542 ± 419), and necrotic cells were normalized to total cell count. The ΔCt and Ct*_Actb_* of (**C**–**F**) are summarized in Supplementary Table S2. The mean ± SEM of biological replicates is shown. Significance was determined using a paired Student’s *t* test. * *p* < 0.05, ** *p* < 0.01.

Since the stimulation of D13 + Insulin cells with different macrophage supernatants did not optimize the cell model due to acute stimulation being unfeasible for secretion assays, we focused on stimulation with factors secreted by macrophages. Here, we decided to only proceed with substances secreted by M1 macrophages, the most prominent ATM in obesity [44,45]. Activated M1 macrophages secrete pro-inflammatory factors like tumor necrosis factor α (Tnfα) [46]. Tnfα serum levels are increased in patients with obesity [47] or type 2 diabetes [47,48], leading to reduced insulin sensitivity [49] and adiponectin secretion [50,51]. In order to induce a more pathophysiologically hypertrophic state in 3T3-L1 adipocytes, insulin-Tnfα co-stimulation appeared to be a promising approach. Therefore, we differentiated 3T3-L1 cells under insulin stimulation until D10, followed by three days of insulin–Tnfα co-stimulation (Figure 5, Supplementary Figure S2).

Low doses of Tnfα (4 ng/mL) did not lead to an alteration in lipid accumulation (Supplementary Figure S2A–E) or cell size (Supplementary Figure S2F–H). Analyzing insulin sensitivity, we detected even further reduced acute insulin-stimulated glucose uptake (Figure 5A) and AKT phosphorylation (Figure 5B) under co-stimulation (D13 + Insulin + Tnfα) compared to D13 + Insulin adipocytes. Again, *InsR* expression was not altered, and *Glut4* mRNA was induced to insufficiently compensate for the reduced insulin sensitivity compared to D10 *w/o* adipocytes (Figure 5C, Supplementary Table S2). Regarding the expression and secretion levels of cytokines and adipokines, *Il6*/IL6 expression and secretion were significantly induced in D13 + Insulin + Tnfα adipocytes compared to D13 + Insulin adipocytes (Figure 5D, Supplementary Table S2), whereas *Lep*/LEP expression and secretion were not changed (Figure 5E, Supplementary Table S2). Also, insulin–Tnfα co-stimulation did not influence *AdipoQ* expression; however, adiponectin secretion was reduced almost to D10 *w/o* levels (Figure 5F, Supplementary Table S2). To investigate if acute insulin and Tnfα stimulation is mandatory, different acute incubation combinations were performed. In this way, we found acute insulin stimulation to dampen Tnfα-increased IL6 secretion (Figure 5D, Supplementary Figure S2I) and to rescue leptin levels (Figure 5E, Supplementary Figure S2J), whereas it had no effect on adiponectin levels (Figure 5F, Supplementary Figure S2K). Therefore, acute insulin is an important factor during starvation and stimulation.

Since Tnfα is involved in apoptosis by inducing the death receptor pathway [52,53], we analyzed adipocyte viability. Here, apoptosis and necrosis were induced in adipocytes co-stimulated with insulin and Tnfα (Figure 5G,H, Supplementary Figure S2L).

**Figure 6:**
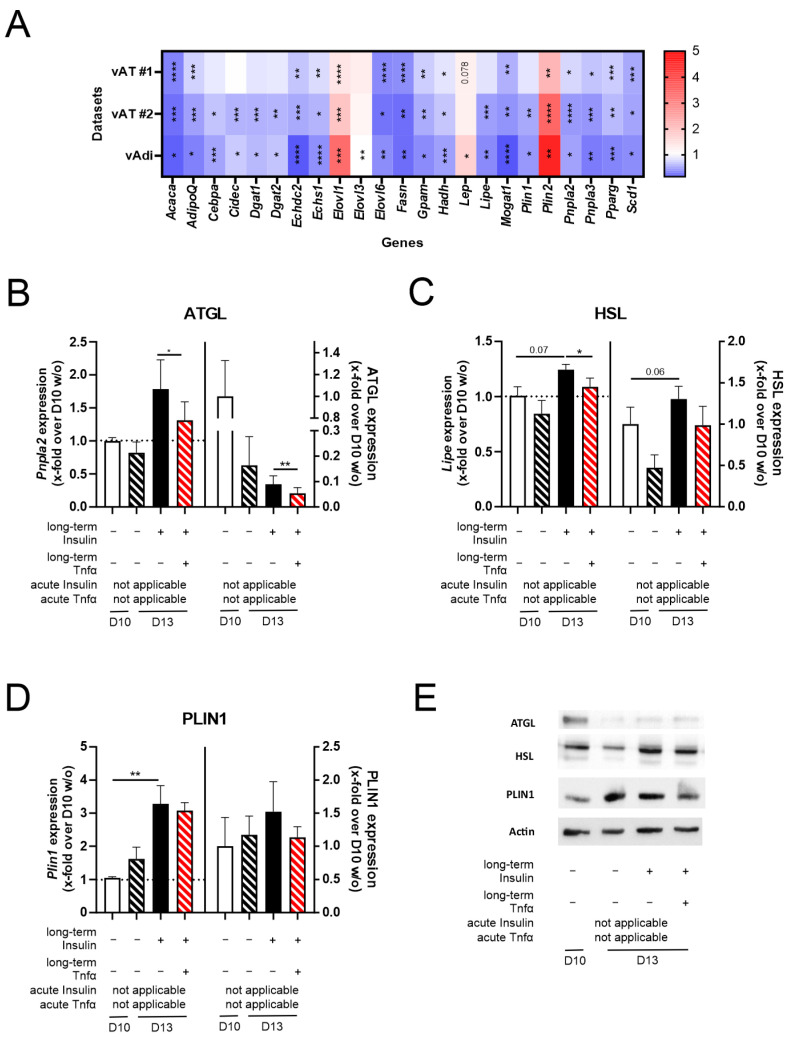
The impact of Tnfα and long-term insulin stimulation on 3T3-L1 adipocytes regarding the expression of additional genes differentially regulated in the adipocytes of lean and obese mice. For further validation of the pathophysiologically hypertrophic characteristics of the cell model (3T3-L1 D13 + Insulin + Tnfα), (**A**) genes involved in lipid deposition, lipolysis, and adipogenesis were investigated in RNAseq datasets of visceral AT and visceral adipocytes (vAdi). Only differentially expressed genes are shown as a ratio of obese/lean. (**B**–**E**) After differentiating 3T3-L1 adipocytes under long-term insulin stimulation until D13 of differentiation, with optional Tnfα stimulation from D10 to D13 (Figure 1), the gene and protein expressions of (**B**) ATGL (*n* ≥ 3), (**C**) HSL (*n* ≥ 3), and (**D**) PLIN1 (*n* ≥ 3) were investigated representatively. One representative Western blot per protein is depicted in (**E**), whole Western blots are provided in Supplementary Figure S3, and protein expression values are given in Supplementary Table S4. Expression values are shown in Supplementary Table S3. As a control, standard differentiated 3T3-L1 cells (D10 *w/o*) were used. Gene expression data were normalized to *Actb* and D10 *w/o*, and protein expression was normalized to β-actin and D10 *w/o*. Given is the mean ± SEM of biological replicates. Significant changes were tested using a paired Student’s *t* test. * *p* < 0.05, ** *p* < 0.01, *** *p* < 0.001, **** *p* < 0.0001.

In the next step, we further evaluated the properties of the established cell model, analyzing its impact on the mRNA and protein expression levels of genes/proteins involved in adipogenesis, lipid deposition, and lipolysis. Therefore, we extracted genes differentially expressed between normal and hypertrophic adipocytes using Chip seq data for normal and hypertrophic adipocytes [54] and compared them with RNAseq datasets of visceral AT and visceral adipocytes for lean individuals and individuals with obesity [32]. We identified 23 genes differentially regulated in both the Chip seq data of adipocytes and the RNAseq data of visceral adipocytes (Figure 6A). During antibody validation, we were only able to identify functional anti-ATGL, anti-HSL, and anti-PLIN1 antibodies and decided to precede with these three genes/proteins. When investigating changes in the gene and protein expression of *Pnpla2*/ATGL, *Lipe*/HSL, and *Plin1*/PLIN1, we found that regulation was similar to in in vivo conditions (Figure 6B–E and Supplementary Table S3).

Taken together, (acute) insulin and Tnfα co-stimulation reinforced the effect of D13 + Insulin on insulin sensitivity and reduced adiponectin secretion almost to D10 *w/o* levels. Further, co-stimulation induced a higher inflammatory state compared to D10 *w/o* adipocytes. Additionally, genes involved in lipid deposition and lipolysis were regulated similar to in vivo conditions.

## 4. Discussion

The rapidly rising prevalence of obesity [1,2] is leading to a high demand for obesity prevention and treatment of affected individuals. Although animal models are an important tool, the high burden on the animals and the time requirements are major downsides. Therefore, both basic research and drug development would greatly benefit from the development of a cell model mimicking pathophysiologic hypertrophy. In obesity, adipose tissue mainly expands by adipocyte hypertrophy instead of adipocyte hyperplasia and adipogenesis [10,11]. Hypertrophic adipocytes do not only show phenotypic alterations (increased cell size and higher lipid accumulation) but also functional alterations (reduced insulin sensitivity [11,12,13] and changes in adipokine and cytokine secretion [11,14]). To mimic these effects in vitro, we established a cell model to analyze adipocytes in a pathophysiologically hypertrophic state using the model cell line 3T3-L1 (Figure 1).

First, we validated published approaches utilizing glucose or FFAs as stimuli (Figure 2). Since glucose is converted via glycolysis, the citrate cycle, and lipogenesis to FFAs, variations in glucose concentration in the supernatant can influence lipid accumulation. Therefore, Jackson et al. proposed a 3T3-L1 model depending on varying glucose concentrations [28]. In our study, we only found slight changes in lipid accumulation at different glucose concentrations (D10 + reduced Glucose, Figure 2). Triacylglycerols are transported by very low-density lipoproteins to AT and are hydrolyzed by lipoproteinlipases to glycerol and FFAs, which are taken up by adipocytes. In individuals with obesity, 450 µM of FFAs has been detected in the circulation, of which 100 µM of palmitate and 110 µM of oleate represent the greatest proportion [55]. Therefore, FFAs (palmitate and oleate) have been used to induce hypertrophy in 3T3-L1 cells in multiple studies [25,26,27]. After treating 3T3-L1 adipocytes with 75–750 µM of palmitate or oleate (Figure 2, D12 + FFA/BSA, only 750 µM palmitate shown), we could not detect an effect mediated by FFAs, although we covered a wide range of physiological and unphysiological FFA concentrations. Interestingly, we were able to show the induction of lipid accumulation by adding the carrier protein BSA alone.

Second, we evaluated new approaches and tested insulin stimulation in combination with long-term incubation (Figure 2 and Figure 3). Insulin is the main driver of glucose uptake into a cell and, thereby, leads to the induction of glycolysis, the citrate cycle, and lipogenesis. Indeed, continuous insulin stimulation (D10 + Insulin) significantly induced lipid accumulation and increased droplet size, which was even more pronounced after long-term insulin incubation (D13 + Insulin, Figure 2).

Since long-term insulin incubation (D13 + Insulin) was an effective approach, we confirmed further characteristics of hypertrophic adipocytes like increased cell size, reduced insulin sensitivity, and induced leptin secretion (Figure 3). However, the inflammatory/pathophysiologic status of hypertrophic adipocytes was not observed.

Inflammation in the AT of individuals with obesity is marked by increased infiltration of ATMs accompanied by a shift in the M1/M2 ratio to an inflammatory state [44,45]. Here, the increased M1 subpopulation induces the secretion of inflammatory factors such as Tnfα, IL6, and MCP1 [46]. Therefore, we decided to simulate D13 + Insulin adipocytes with a macrophage supernatant. RAW264.7 cells are a widely used mouse monocyte cell line isolated from a virus-induced tumor. Since replication-competent ecotropic and polytropic viruses were detected in the supernatant of RAW264.7 cells [56], a cell model with RAW264.7 supernatant would not be adaptable to every laboratory and, therefore, its usage would be restricted to a limited group of researchers. Therefore, we searched for an alternative cell line. The human macrophage cell line THP1 has been used for co-culture experiments with 3T3-L1 cells previously [57]. Since the secretion profiles of human (THP1) and mouse (RAW264.7) macrophages are similar [58] and human cytokines are able to bind to mouse receptors [59,60,61], we decided to continue with THP1-3T3-L1 experiments (Figure 4, Supplementary Figure S1). When evaluating the influence of the supernatants of the M0, M1, and M2 macrophage subpopulations on D13 + Insulin adipocytes, no changes in any of the analyzed phenotypic or functional characteristics of D13 + Insulin adipocytes were observed (Figure 4, Supplementary Figure S1). However, the effects mediated by stimulation with the macrophage supernatant might not be detectable in secretion studies, since the starvation and stimulation of 3T3-L1 adipocytes needed to be carried out without an acute macrophage supernatant stimulation. Serum starvation induces a high amount of stress in THP1 macrophages, leading to a substantial loss in viability. Collecting the M0, M1, and M2 macrophage supernatants of these starved THP1 macrophages may have led to a different secretion pattern prone to a loss in 3T3-L1 adipocyte viability. Further, the various proteins secreted by macrophages might interfere with the secretion analysis of 3T3-L1 cells. In particular, M1 macrophages secrete inflammatory factors like IL6 [46], which would complicate the detection of the inflammatory status of 3T3-L1 adipocytes. Therefore, the acute effects of the THP1 macrophage supernatant on 3T3-L1 adipocytes could not be analyzed.

Stimulation with a recombinant protein leads to results with higher reproducibility compared to the supernatant of cells, whose composition depends on cellular fitness. Therefore, stimulation with a recombinant protein simplifies a cell model. To improve our preliminary cell model, we decided to stimulate D13 + Insulin adipocytes with the recombinantly expressed pro-inflammatory cytokine Tnfα to induce an inflammatory state (Figure 5, Supplementary Figure S2). Tnfα is secreted by pro-inflammatory M1 macrophages, the most prominent ATMs in obesity [44,45]. Further, Tnfα is negatively correlated with insulin sensitivity [49] and adiponectin secretion [50,51]. Indeed, the long-term and acute Tnfα–insulin stimulation of adipocytes induced IL6 expression and decreased adiponectin secretion. Interestingly, acute stimulation with Tnfα alone had a negative effect on leptin secretion. Since insulin stimulates leptin secretion [62,63], co-stimulating acutely Tnfα-treated adipocytes with acute insulin was necessary to restore leptin levels. Moreover, acute co-stimulation was also necessary to dampen IL6 secretion mediated by insulin’s anti-inflammatory characteristics [64,65,66]. Furthermore, we detected an upregulation of apoptosis and necrosis upon acute Tnfα–insulin co-stimulation. Adipocyte hypertrophy and M1 macrophages are connected to increased apoptosis [67,68], which can be mimicked through Tnfα stimulation of adipocytes [69,70]. Due to apoptotic adipocytes not being cleared, these cells undergo secondary necrosis [67]. To further validate our cell model, we identified genes differentially regulated in obesity and involved in lipolysis, lipid deposition, and adipogenesis and reproduced the regulation of these genes/proteins in our cell model. Here, we observed the expected downregulation of the lipolytic enzymes ATGL and HSL (Figure 6).

In summary, we developed a cell model of hypertrophic adipocytes using the 3T3-L1 cell line. Here, insulin and Tnfα co-stimulation in combination with prolonged incubation led to hypertrophic changes in adipocyte phenotype and function. We were able to increase cell size, reduce insulin sensitivity, change the protein expression of lipases and perilipins, and alter secretion patterns (increased leptin and IL6 and reduced adiponectin) to reach a pathophysiological state.

## 5. Conclusions

In conclusion, our study supplies an in vitro cell model for analyzing the pathophysiological state of adipocyte hypertrophy under obese conditions. This hypertrophic cell model can easily be used to analyze the effects of knockdown, overexpression, or stimulation studies. In future studies, this 2D cell model could be further developed into a 3D approach to achieve conditions comparable to in vivo models. Additionally, the model could be made applicable to human cell lines like SGBS cells.

## Figures and Tables

**Table 1:** 3T3-L1 cells were differentiated under various conditions to generate (patho)physiologically hypertrophic 3T3-L1 adipocytes. CM: adipocyte culture media (DMEM (5.55 mM or 25 mM glucose) + 10% FBS + 1°% penicillin/streptomycin); concentrations of reagents were as follows: 0.172 mM insulin, 0.25 µM dexamethasone, 0.5 mM IBMX, 2 µM rosiglitazone, 4 ng/mL Tnfα (Thermo Fisher Scientific, Waltham, MA, USA), and 75–750 µM of FFAs (palmitate (Sigma-Aldrich, St. Louis, MO, USA) or oleate (Sigma-Aldrich, St. Louis, MO, USA).

	Days of Differentiation				
Approach	Day 0–Day 3	Day 3–Day 6	Day 6–Day 10	Day 10–Day 12	Day 12–Day 13
Control	CM + Dexamethason + IBMX + Insulin + Rosiglitazone	CM + Insulin	CM	-	-
I (Reduced Glucose)	CM + Dexamethason + IBMX + Insulin + Rosiglitazone	CM (Reduced Glucose) + Insulin	CM (Reduced Glucose) + Insulin	-	-
II (long-term incubation)	CM + Dexamethason + IBMX + Insulin + Rosiglitazone	CM + Insulin	CM	CM	-
III (FFAs)	CM + Dexamethason + IBMX + Insulin + Rosiglitazone	CM + Insulin	CM	CM + FFAs	-
IV (Insulin)	CM + Dexamethason + IBMX + Insulin + Rosiglitazone	CM + Insulin	CM + Insulin	-	-
V (long-term incubation + Insulin)	CM + Dexamethason + IBMX + Insulin + Rosiglitazone	CM + Insulin	CM + Insulin	CM + Insulin	CM + Insulin
VI (long-term incubation + Insulin + THP1 supernatant)	CM + Dexamethason + IBMX + Insulin + Rosiglitazone	CM + Insulin	CM + Insulin	CM + Insulin + THP1 M0, M1, or M2 macrophage supernatant	CM + Insulin + THP1 M0, M1, or M2 macrophage supernatant
VII (long-term incubation + Insulin + Tnfα)	CM + Dexamethason + IBMX + Insulin + Rosiglitazone	CM + Insulin	CM + Insulin	CM + Insulin + Tnfα	CM + Insulin + Tnfα

## Data Availability

Data are contained within the article or Supplementary Material. The original contributions presented in this study are included in the article/Supplementary Material. Further inquiries can be directed to the corresponding author.

## Supplementary Materials

The following supporting information can be downloaded at: https://www.mdpi.com/article/10.3390/cells14110837/s1, Supplementary Figure S1: Variations in lipid accumulation, cell size and viability of 3T3-L1 adipocytes after long-term insulin stimulation in combination with M0, M1, and M2 THP1 macrophage supernatant. Supplementary Figure S2: The effect of insulin and Tnfα co-stimulation on the lipid accumulation, cell size, and viability of 3T3-L1 adipocytes. Supplementary Figure S3: Western blots for protein expression analysis depicted in Figure 6. Supplementary Table S1: Sequences of qPCR primers. Supplementary Table S2: Gene expression levels described in Figure 3, Figure 4 and Figure 5. Supplementary Table S3: Gene expression levels described in Figure 6. Supplementary Table S4: Protein expression levels described in Figure 6 and Supplementary Figure S3. Protein expression for indicated proteins was detected. Supplementary zip file: The original images displayed in the main text and Supplementary Material.
