# Supplementary material for: A Pathophysiologically Hypertrophic 3T3-L1 Cell Model—An Alternative to Primary Cells Isolated from DIO Mice
Source: Cells. 2025 Jun 3;14(11):837. doi: 10.3390/cells14110837 (PMC12155249; doi:10.3390/cells14110837)
Supplement: Supplementary file 1 [file cells-14-00837-s001.zip › cells-3656490-supplementary.pdf]

A pathophysiologically hypertrophic 3T3-L1 cell model– an al-ternative to isolated primary cells from DIO mice

Isabell Kaczmarek[1], Kristiana Schüßler[1], Andreas Lindhorst[2], Martin Gericke[2], Doreen Thor1[*]

1 Rudolf Schönheimer Institute of Biochemistry, Medical Faculty, Leipzig University, 04103 Leipzig, Germany
2 Institute of Anatomy, Medical Faculty, Leipzig University, 04103 Leipzig, Germany.
* Correspondence: doreen.thor@medizin.uni-leipzig.de

Supplementary File

Supplementary Table S1: Sequences of qPCR primers

		Sequence	
Gene	Protein	sense	antisense
InsR	INSR	GGAATGTGGGGATGTCTGTCC	CTGTGCAGCCATGTGACTTA
Glut4	GLUT4	GGTGTGGTCAATACGGTCTTCAC	AGCAGAGCCACGGTCATCAAGA
Il6	IL6	TCCGGAGAGGAGACTTCACA	TTCTGCAAGTGCATCATCGT
Lep	LEP	GCAGTGCCTATCCAGAAAGTCC	GGAATGAAGTCCAAGCCAGTGAC
AdipoQ	ADIPO	AGTGGATCTGACGACACCAAA	TCATCTTCGGCATGACTGGG
Pnpla2	ATGL	AGTTGTCCCCCAGGAAGAGGAT	AGGGTGGTCATCAGGTCCTT
Lipe	HSL	CATCAACCGACCAGGAGTGCT	GCAGCCTTTGTGTAGCGTGA
Plin1	PLIN1	GAGAAGGTGGTAGAGTTCCTCC	GTGTGTCGAGAAAGAGTGTTGGC
Actb	B-Actin	GCTCTTTTCCAGCCTTCCTT	CGGATGTCAACGTCACACTT

Supplementary Table S2: Gene expression levels described in Figures 3, 4, and 5. Gene expression for indicated genes was detected. ΔCt values were calculated using *Actb* as house keeping gene.

		ΔCt										Ct	
		InsR		*Glut4*		*Il6*		*Lep*		*AdipoQ*		*Actb*	
	Condition	mean	SEM	mean	SEM	mean	SEM	mean	SEM	mean	SEM	mean	SEM
Figure 3	D10 w/o	5.28	0.28	4.46	0.30	10.70	0.67	10.09	0.84	-1.66	0.84	15.52	0.23
	D10+Insulin	5.07	0.23	2.96	0.13	12.19	0.61	9.30	0.97	-1.88	0.97	15.89	0.18
	D13 w/o	5.42	0.29	4.65	0.19	10.56	0.32	8.90	1.01	-1.71	1.01	15.26	0.24
	D13+Insulin	5.28	0.25	3.23	0.20	11.28	0.28	6.35	1.11	-1.99	1.11	15.85	0.20
Figure 4	D10 w/o	4.95	0.29	3.65	0.28	10.65	0.23	6.99	0.67	-0.75	0.24	15.79	0.24
	D13+Insulin	4.93	0.27	2.90	0.28	10.21	0.08	5.64	0.42	-0.89	0.28	15.84	0.28
	D13+Insulin+M0	5.95	0.32	3.93	0.33	9.42	0.06	6.61	0.39	2.00	0.25	15.00	0.25
	D13+Insulin+M1	5.11	0.26	3.28	0.21	9.62	0.19	6.10	0.39	0.58	0.34	15.60	0.34
	D13+Insulin+M2	4.95	0.17	2.89	0.29	9.60	0.11	5.82	0.48	-0.42	0.14	15.76	0.14
Figure 5	D10 w/o	5.13	0.17	4.63	0.23	11.46	0.66	10.09	0.33	-1.66	0.84	15.44	0.30
	D13+Insulin	5.16	0.20	3.19	0.24	11.45	0.39	6.35	0.19	-1.99	1.11	15.80	0.22
	D13+Insulin+Tnfα	5.45	0.10	3.54	0.17	9.76	0.55	6.83	0.17	-1.44	0.72	15.56	0.22

Supplementary Table S3: Gene expression levels described in Figure 6. Gene expression for indicated genes was detected. ΔCt values were calculated using *Actb* as house keeping gene.

	ΔCt						Ct	
	Pnpla2		*Lipe*		*Plin1*		*Actb*	
Condition	mean	SEM	mean	SEM	mean	SEM	mean	SEM
D10 w/o	2.05	0.10	1.87	0.17	6.06	0.18	15.95	0.14
D13+Insulin	1.34	0.32	1.62	0.09	4.45	0.31	16.32	0.22
D13+Insulin+Tnfα	1.76	0.27	1.82	0.08	4.46	0.19	16.03	0.21

Supplementary Table S4: Protein expression levels described in Figure 6 and Supplementary Figure S3. Protein expression for indicated proteins was detected.

	ATGL/β-Actin		HSL/β-Actin		PLIN1/β-Actin		β-Actin	
Condition	mean	SEM	mean	SEM	mean	SEM	mean	SEM
D10 w/o	0.162	0.053	0.064	0.013	0.113	0.049	96070774	15468566
D13 w/o	0.025	0.019	0.033	0.015	0.125	0.068	84274933	21264027
D13+Insulin	0.011	0.002	0.079	0.010	0.128	0.033	85881506	11572221
D13+Insulin+Tnf$\alpha\alpha$	0.006	0.002	0.068	0.025	0.113	0.039	67293358	3560777

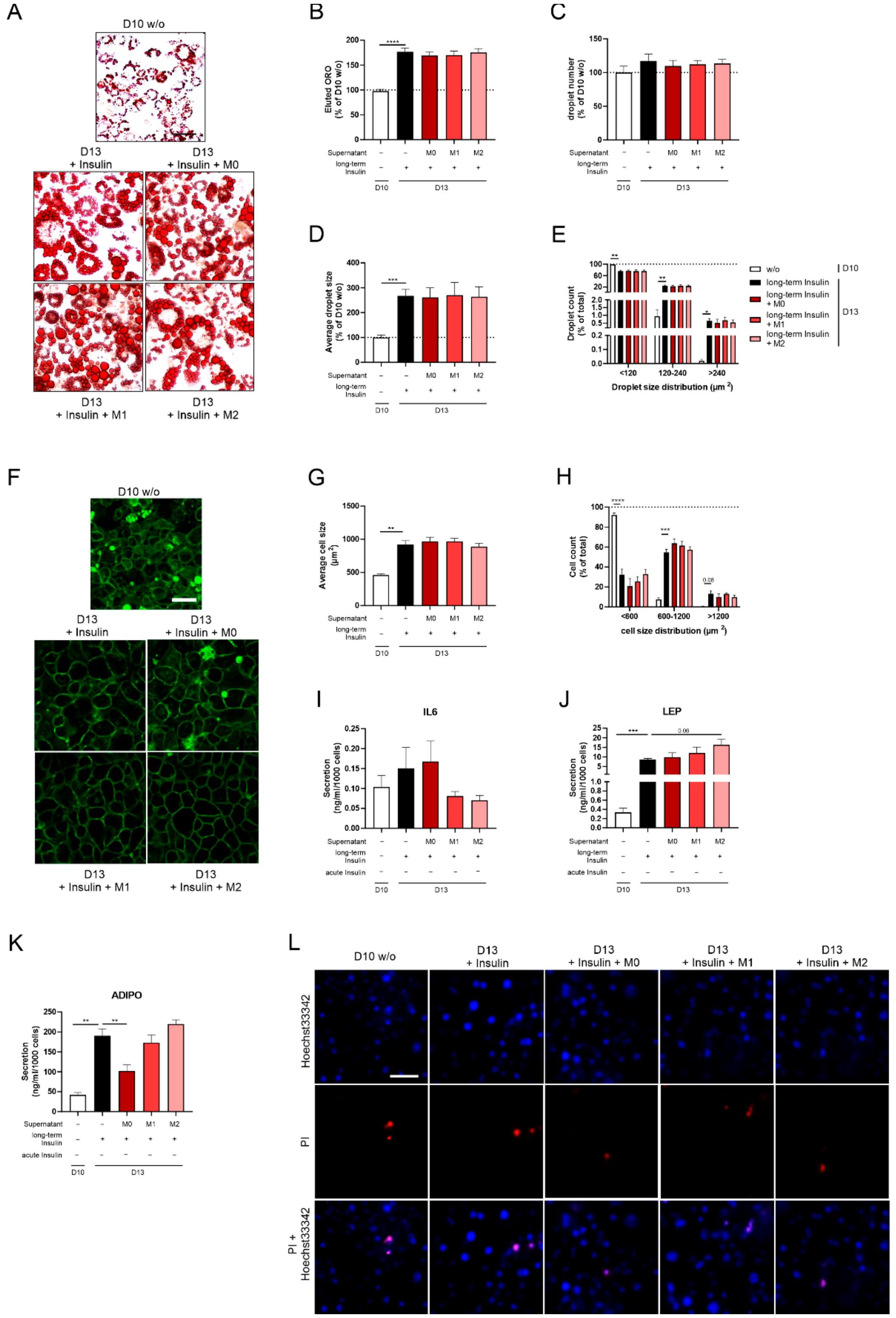

Supplementary Figure S1: Variations in lipid accumulation, cell size and viability of 3T3-L1 adipocytes after long-term insulin-stimulation in combination with M0-, M1-, and M2-THP1 macrophage supernatant. 3T3-L1 were differentiated until D10 or D13 with an optional stimulation of insulin and THP1-macrophage supernatant. As control standard differentiated 3T3-L1 (D10 w/o) were used. To evaluate lipid accumulation, A) microscopic pictures were taken after ORO stain (scale bar: 50 µm) before quantitative and qualitative lipid droplet analysis. B) Lipid accumulation was quantified by eluting ORO. Data were normalized to D10 w/o ($OD_{500\,nm-620\,nm} = 0.42 \pm 0.01$, n=7). C) Droplet number (n≥5), D) average droplet size (n≥6) and E) lipid droplet size distribution (n≥5) were detected. Droplet number and average droplet size were normalized to D10 w/o ($n_{droplet} = 5965 \pm 569$, $A_{droplet} = 19.3 \pm 1.8\ \mu m^2$). For cell size analysis F) microscopic pictures of differently treated 3T3-L1 cells were taken after Cell Mask staining (scale bar: 50 µm) to determine G) the average cell size (n=4) and H) cell size distribution (n=4). To

validate the effect of insulin on secretion (compare to Figure 4D-F), secretion of I) IL6, J) LEP and K) ADIPO were detected in adipocytes starved and stimulated in serum-free media without insulin as indicated (acute insulin). Secretory levels were normalized to cell count. L) Microsopy of adipocytes treated with or without insulin/ macrophage supernatant was used to count necrotic and total cell number (compare to Figure 4H, scale bar: 50 μm). Given is the mean ± SEM of biological replicates. Significant changes were tested using a paired Student's t test (B-D, G, I-K) and two-way ANOVA (E, H). $*p < 0.05$, $**p < 0.01$, $***p < 0.001$, $****p < 0.0001$.

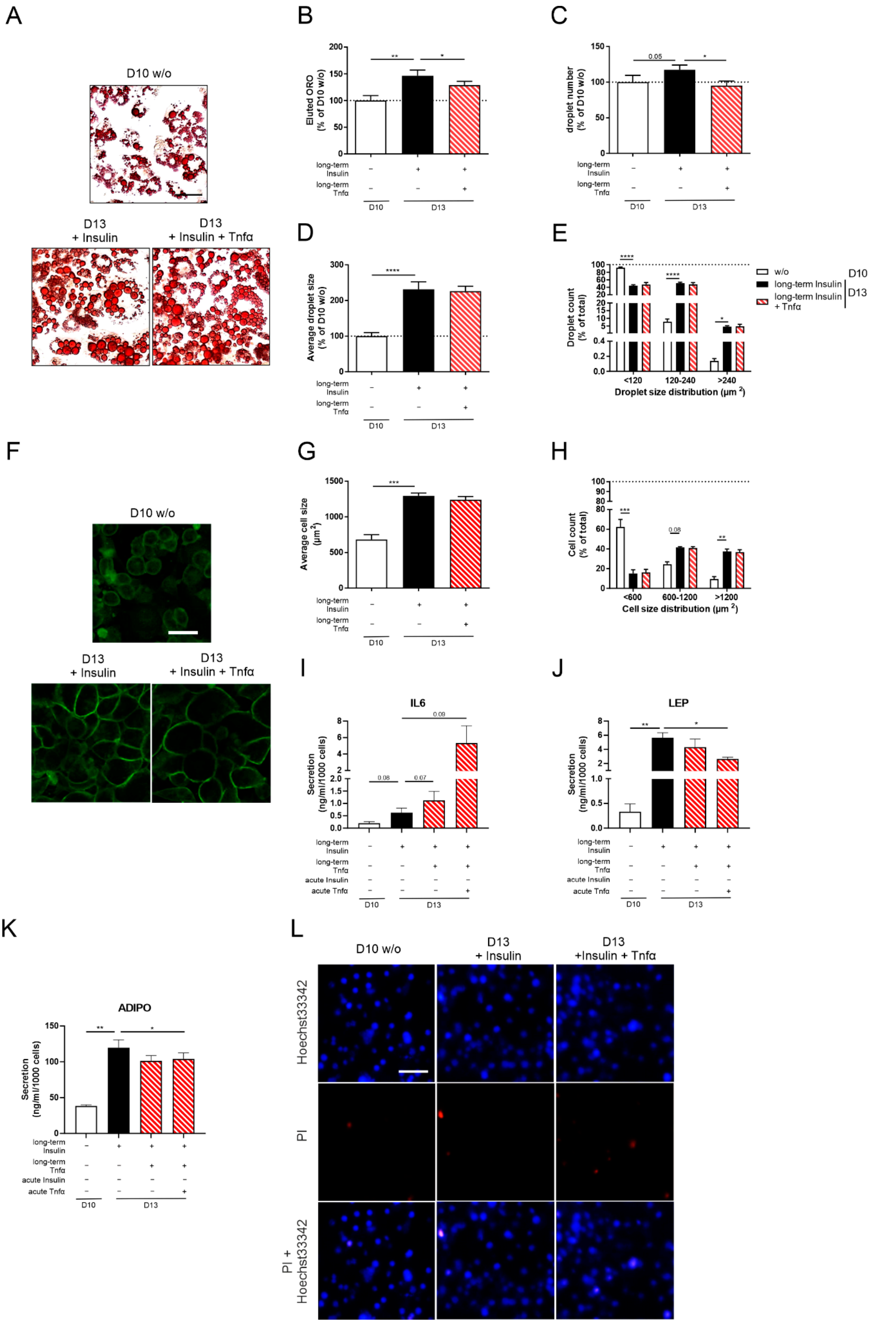

Supplementary Figure S2: The effect of insulin and Tnfα co-stimulation on lipid accumulation, cell size, and viability of 3T3-L1 adipocytes. 3T3-L1 were differentiated until D10 or D13 in the presence or absence of insulin and Tnfα (Figure 1). As control standard differentiated 3T3-L1 (D10 w/o) were used. To investigate lipid droplets,

A) microscopic pictures were taken (scale bar: 50 µm) and B) lipid accumulation was quantified eluting ORO (n=6). For qualitative analysis, C) lipid droplet number (n=6), D) average droplet size (n=6) and E) droplet size distribution (n=6) were analyzed. Data in B)-D) were normalized to D10 w/o ($OD_{500\,nm-620\,nm}$ = 0.49 ± 0.04, $n_{droplet}$ = 4442 ± 421, $A_{droplet}$ = 36.7 ± 3.5 µm^2). Evaluating cell size, F) microscopic pictures were taken (scale bar: 50 µm) and G) average cell size (n=5) as well as H) cell size distribution (n=5) were measured. To validate the effect of insulin and Tnfα on secretion (compare to Figure 5D-F), secretion of I) IL6, J) LEP and K) ADIPO were detected in adipocytes starved and stimulated in serum-free media without insulin (acute Insulin) and with / without Tnfα supplementation (acute Tnfα) as indicated. Secretory levels were normalized to cell count. L) Cells incubated with or without insulin or Tnfα were microscopically analyzed to detect necrotic cells and total cell count (compare to Figure 5H, scale bar: 50 µm). Given is the mean ± SEM of biological replicates. Significant changes were tested using a paired Student's t test (B-D, G, and I-K) and two-way ANOVA (E and H). *$p < 0.05$, **$p < 0.01$, ***$p < 0.001$, ****$p < 0.0001$.

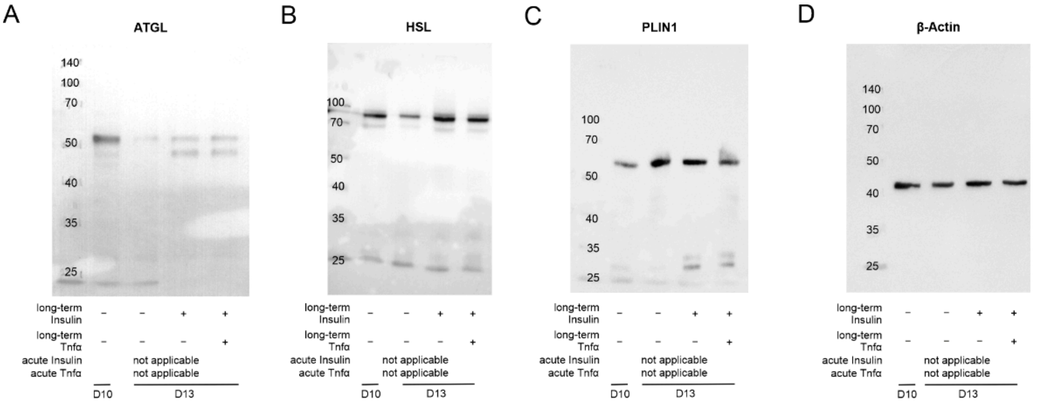

Supplementary Figure S3: Western Blots for protein expression analysis depicted in Figure 6. 3T3-L1 were differentiated until D10 or D13 in the presence or absence of insulin and Tnfα (Figure 1). As control standard differentiated 3T3-L1 (D10 w/o) were used. Protein expression was deteced for A) ATGL, B) HSL, C) PLIN1, and D) β-Actin. The shown Western Blots depict one representative, biological replicate. Protein expression values are shown in Supplementary Table S4.
